# Mapping definitions and measures of police violence in health literature: a scoping review

**DOI:** 10.1186/s12889-026-28146-x

**Published:** 2026-06-18

**Authors:** Latesha K. Harris, Jamie L. Conklin, Valentina Marginean, Cheryl Woods Giscombé, Yamnia I. Cortés

**Affiliations:** 1 School of Nursing and National Clinician Scholars Program, Duke University, 307 Trent Dr Durham, Durham, NC 27710 USA; 2 Health Science Library, The University of North Carolina at Chapel Hill, 335 S. Columbia Street, CB 7585, Chapel Hill, NC 27599-7585 USA; 3 College of Nursing, University of Iowa, 50 Newton Rd, Rm 454 CNB, IA 52242 Iowa City, USA; 4 School of Nursing, The University of North Carolina at Chapel Hill, Carrington Hall, CB 7640, Chapel Hill, NC 27599-7460 USA

**Keywords:** Police violence, Police brutality, Use of force, Public health, Health policy, Health inequities, Scoping review

## Abstract

**Background:**

Police violence continues to be a significant public health problem, especially in the United States. A limited but growing body of research has explored the relationship between police violence and health outcomes. Emerging evidence suggests that exposure to police violence may be a contributing factor to health inequities, particularly among Black Americans. Yet, there remains a lack of consensus on a conceptual and operational definition of police violence within health disciplines. This scoping review aims to address this gap by mapping the definitions and measures for assessing police violence in health literature.

**Methods:**

We comprehensively searched PubMed, Cumulative Index to Nursing and Allied Health Literature (CINAHL), and APA PsycInfo databases. We included studies published as peer-reviewed primary research in health journals and in the English language. Eligible studies measured police violence.

**Results:**

Of the 1692 references identified, 204 references were retained for full-text review, and 114 references reporting on 98 studies met the inclusion criteria. We identified over 100 terms for police violence, which we clustered into 22 categories including abuse, assault, harassment, killing, and violence. The most common terms were “police violence,” “police abuse,” and “police brutality.” Only two-thirds of studies (*n* = 76) included a definition of police violence. Police violence was most frequently defined as “police killings” (*n* = 23) and excessive use-of-force (*n* = 10). Self-reports of police violence were the most common measure (*n* = 48). Other measures included counts of fatal police violence from open-source and governmental databases, as well as counts of deaths/injuries classified as “legal intervention” in medical records.

**Conclusions:**

The health literature lacks a clear and consistent definition of police violence. While multiple measures show some potential for surveillance of police violence, few studies have conducted in-depth psychometric analyses. Recommendations for future research include developing an explicit definition of police violence and reporting psychometric data for measures. Additionally, there is a need for systematic data collection of police violence in health settings and the establishment of a federally supported and accessible public database of police violence to address underreporting and undermeasurement of police violence.

## Background

Police violence, a significant public health issue in the United States (U.S.) [1–3], remains challenging to define and quantify [4]. Ambiguities about what constitutes police violence, particularly whether it refers only to excessive police use of force or also to other harmful or discriminatory policing practices, contribute to divergent definitions, measurement challenges, and inconsistent prevalence estimates in the U.S. Although the U.S. makes up only 4% of the world’s population, it accounts for nearly 13% of global fatal police-related deaths [5]. While police violence is inconsistently measured and underreported [4], available data demonstrate a pronounced racial difference in the risk of death after a police encounter. For example, the risk of death after a police encounter in the U.S. is 2.5 times higher for African American/Black men compared to White men [6] and 1.4 times higher for African American/Black women compared to White women [7]. Public health and human rights concerns regarding police violence have been intensified by the police killings of George Floyd, Breonna Taylor, Ta’Kiya Young, Tyre Nichols, Michael Brown, Philando Castille, and many others [8–11]. Similar concerns have been documented globally including high-profile cases in Brazil, Nigeria, France, and South Africa [12–15].

The American Public Health Association has identified police violence as a public health priority [16, 17]. However, there is little consensus on measuring or defining police violence. In fact, few studies even include a definition of police violence and use various methods to measure it [18–22]. The World Health Organization (WHO) broadly defines violence as the “intentional use of physical force or power, threatened or actual, against oneself, another person, or against a group or community, that either results in or has a high likelihood of resulting in injury, death, psychological harm, maldevelopment, or deprivation” [21]. While the WHO definition provides a broad framework encompassing both the use of physical force and the exercise of power that results in psychological harm, we recognize that not all police encounters involving authority constitute “violence.” In this scoping review, we focus specifically on encounters that constitute police misconduct (e.g., racial profiling, unlawful searches, false arrest), or coercive harmful uses of authority that may legally be permissible but are experienced as threatening or discriminatory (e.g., excessive traffic stops, frisking), whether physical violence was present or not. This approach may capture a wider spectrum of experiences than studies using narrower, force-only definitions. This is consistent with the purpose of scoping reviews, which is to map heterogeneity rather than adjudicate a single operational definition [23, 24].

A limited but growing body of research has examined the link between police interactions and health outcomes. Two recent systematic literature reviews [25, 26] have found that police exposure (e.g., indirect experiences with police such as witnessing or hearing stories of police) and police interactions (e.g., direct contact with police officers) may negatively impact the mental health and health behaviors of African Americans. Findings suggest that negative police encounters may be associated with suicidal ideation, psychological distress, and post-traumatic stress disorder [27–29]. Police brutality has also been linked to non-fatal injuries, death, stress, and financial strain [30]. Beyond physical and psychological harm, recent evidence suggests that negative encounters with the police may be associated with a greater medical mistrust, and in turn higher reported unmet health needs [31]. These findings suggest that police violence functions as a social determinant of health, influencing access to care and health behaviors. These findings highlight the importance of further exploring how police violence may affect health outcomes. Although there is a pressing need for deeper exploration of police violence, a standardized definition of the term is lacking. Additionally, current health literature does not present a standardized approach for measuring police violence.

Several academic disciplines such as sociology [32], criminology [33–35], and more recently, public health, have proposed definitions and frameworks for understanding police violence [32, 36, 37]. However, little is known about how police violence has been defined and measured within the health literature itself. Unlike conceptual or theoretical papers, the present scoping review does not aim to evaluate existing frameworks or propose a new one. Instead, its purpose is to identify and map the definitions and measurement approaches in health research to inform future studies examining associations between police violence and health. Although a unified conceptual definition of police violence may ultimately support more consistent measurement, developing such a definition lies outside the scope of this review. To our knowledge, no published synthesis has examined how police violence is operationalized in health studies. Therefore, the objective of this scoping review is to describe how police violence has been defined and measured in health literature.

## Methods

This scoping review was conducted following the Preferred Reporting Items for Systematic Reviews and Meta-Analyses extensions for Scoping Reviews [38]. Scoping methodology was best suited for this review because it allows researchers to describe the breadth, range, and nature of existing evidence rather than evaluating or developing conceptual frameworks. Aligned with this purpose, our review aims to identify and map the wide range of terms used to describe police violence (e.g., police brutality, excessive use of force, fatal police encounters), and to catalogue the diverse methods used to measure police violence within the health literature. This descriptive approach is consistent with established scoping review guidance, which focuses on summarizing existing evidence [23, 24]. The study protocol has been previously published elsewhere [39].

### Search strategy and study selection

A health sciences librarian (JLC) was consulted to create a search strategy [39]. A systemic search was conducted using three health sciences databases: PubMed, Cumulative Index to Nursing and Allied Health Literature (CINAHL) Plus with Full Text (EBSCO*host*), and APA PsycInfo (EBSCO*host*). Databases were searched from their dates of inception through the last search date of January 3, 2023. The following is the full search used in PubMed: "police violence"[tiab] OR "police brutality"[tiab] OR "police use of force"[tiab] OR "law enforcement violence"[tiab] OR "law enforcement brutality"[tiab] OR "law enforcement use of force"[tiab] OR “police abuse”[tiab] OR “law enforcement abuse”[tiab] OR (("legal intervention"[tiab] OR "legal interventions"[tiab] OR “excessive force”[tiab: ~ 5]) AND (police[tiab] OR "law enforcement"[tiab])). A hand search of the reference lists of included articles was conducted to retrieve any additional pertinent references missed by the electronic search. All references were imported into EndNote to remove duplicates. The remaining references were uploaded into the Covidence systematic review software (Veritas Health Innovation, Melbourne, Australia) for screening and data charting.

Studies were included in the scoping review if they 1) were published in English, 2) were published as primary research and peer-reviewed, 3) were published in health-related journals, and 4) measured police violence. Studies that measured police violence were included whether or not they had a definition of police violence. Qualitative studies that identified police violence as a key theme were included in the review. Studies that evaluated predictors or perceptions of police violence, violence against police officers, or violence in communities but did not specifically define or measure police violence were excluded. Since the focus of the review was how police violence was defined and measured in original research articles, we excluded commentaries and literature reviews.

**Fig. 1:**
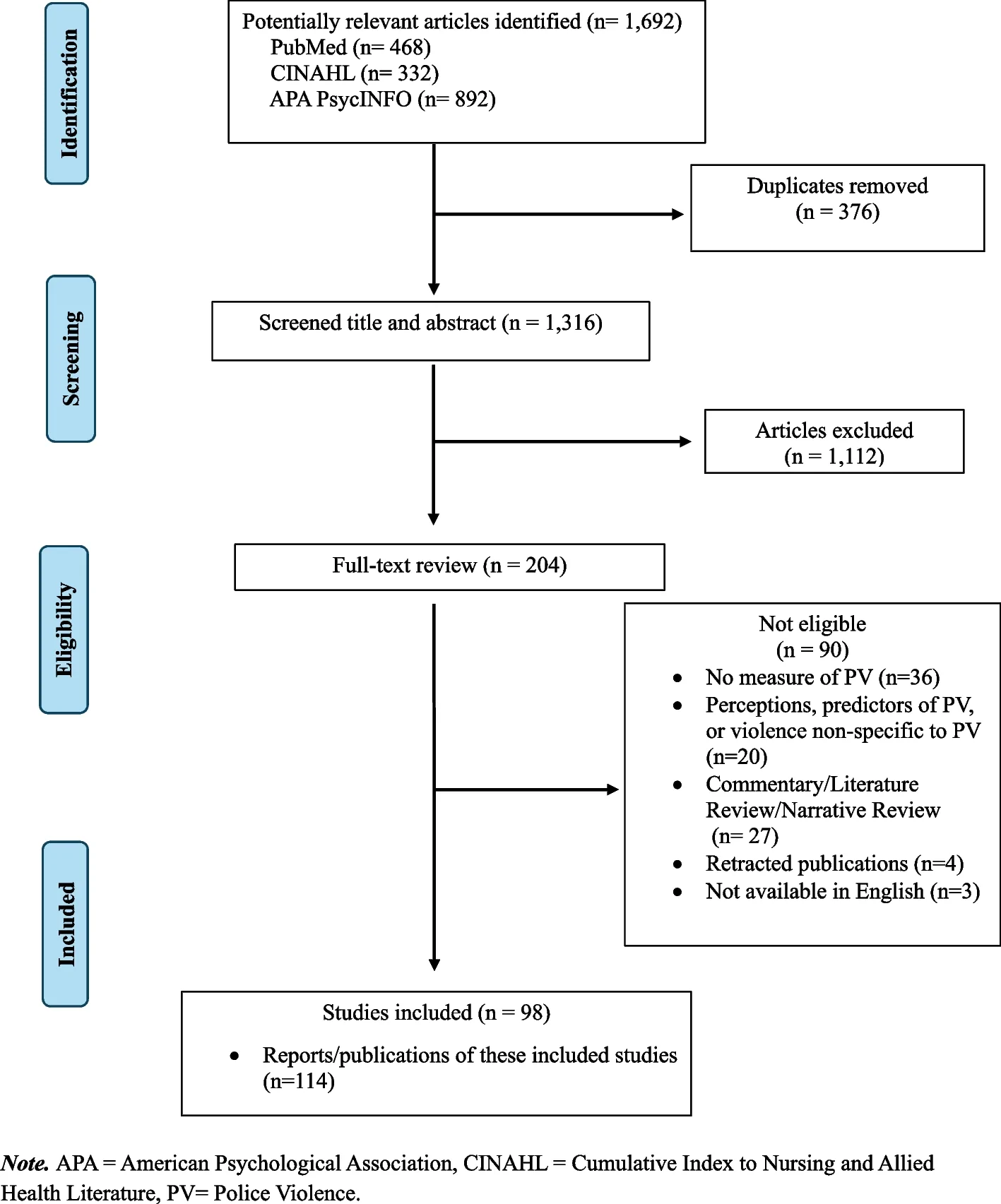
Preferred reporting items for scoping reviews (PRISMA ScR) flow diagram of literature screening and inclusion

Following a pilot screening of a subset (*n* = 30) of references, the titles and abstracts of potentially relevant studies were screened by three researchers (LKH, JLC, YIC) using the inclusion and exclusion criteria. Each title and abstract were independently screened by two of the reviewers for inclusion. Two independent reviewers further reviewed the full-text (LKH, JLC), and those that did not meet the inclusion criteria were excluded (reasons provided in Fig. 1). Disagreements between reviewers were resolved through discussion or with consensus from a third reviewer. Covidence provided the Cohen’s Kappa coefficient for full text review, which was 0.68, indicating substantial agreement (0.61 −0.80) between reviewers [40].

### Charting

Two independent reviewers used a pre-defined charting form to extract relevant data from the included studies [41]. The extracted data included year of publication, study design, study location, population, setting, key terms and concepts of police violence, measures of police violence, and type of health literature or academic discipline where the study was published. Any disagreements between the reviewers on data extraction were resolved through discussion or with a third reviewer. The selected studies were then organized according to the desired outcomes – (1) language encapsulating police violence, (‘*definitions*’), and (2) measures of police violence.

## Results

Figure 1 provides an overview of the selection process for the scoping review. The literature search yielded 1316 references after duplicates were removed. During the title and abstract screening, 1112 references were excluded. Of the 204 identified references, 36 were excluded for not measuring police violence. An additional 20 were excluded because they focused on violence against police officers, general violence non-specific to police, and use of force that was not excessive. A total of 27 commentaries, literature reviews, and editorials were excluded. Next, three references were not available in English and four were retracted. Thus, we included 114 references reporting on 98 unique studies in the review.

While reviews typically combine multiple reports (such as two articles reporting findings on a single study), we have opted to provide report-level findings for publication dates, health focus areas, and police violence terms. This allowed us to provide a more complete analysis as reports provided different police violence terms and were published in different health focus areas. Although the health focus area and terminology for police violence vary across reports, the measurement approach remains consistent because it was defined at the study level. Therefore, we present study-level findings for police violence measurement. In the following results, we clarified the unit of analysis by using the terms reports and studies.

### Study characteristics

**Fig. 2:**
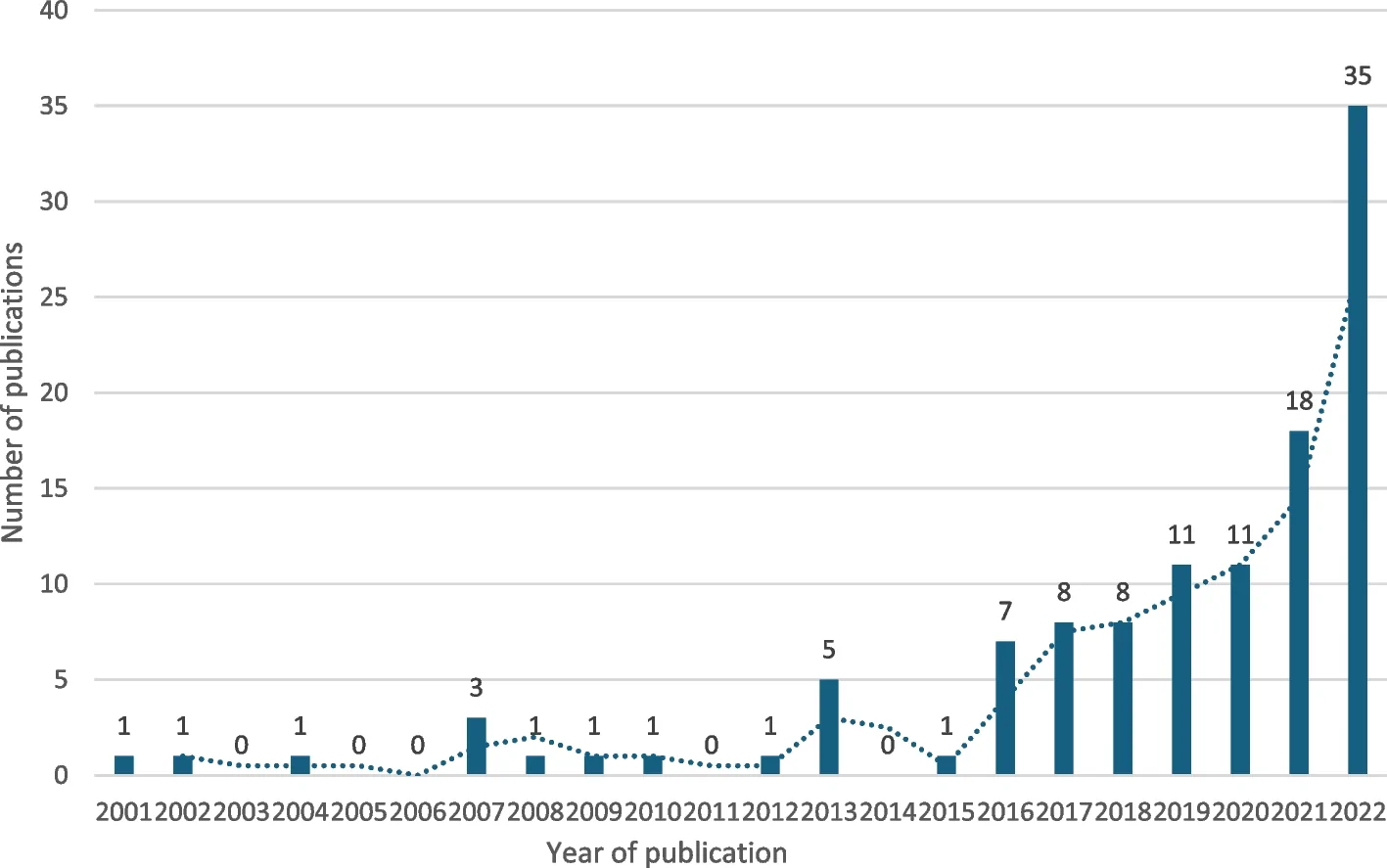
Articles by Publication Year

Figure 2 illustrates the annual number of publications related to police violence that were included in this review and were published in health journals over the past 20 years. The first report was published in 2001, with annual publications ranging from 0 to 5 until 2016 when there were 7 reports. Most reports were published between 2016 and 2022 (*n* = 98; 86%), with the largest number published in 2022 (*n* = 35).

Table 1 presents study characteristics such as location, study design, and sample size. The 98 included studies were conducted in 16 countries, with the majority (*n* = 72; 73%) taking place in the U.S. The studies used various methodological approaches, including qualitative (*n* = 15), quantitative (*n* = 76), and mixed methods (*n* = 7). Among qualitative studies, the majority (*n* = 10) were descriptive in design, reporting on experiences of police violence among youth, sex workers, and sexual and gender minorities. Almost two-thirds (*n* = 48) of quantitative studies were cross-sectional in design, using a variety of public databases such as Mapping Police Violence, National Violent Death Reporting System, and Fatal Encounters. The sample size in each study greatly varied, ranging from 7 to 92,386, with most studies (*n* = 28) including 100–1000 individuals.

**Table 1:** Characteristics of included studies

Study Characteristics	Sample (N)
Country	
Argentina	1
Bolivia	1
Brazil	2
Canada	3
France	2
Hong Kong	1
Indonesia	1
Kazakhstan	1
Kenya	2
Mexico	4
Russia	1
Thailand	4
Turkey	1
UK	1
Ukraine	1
U.S	72
Study Design	
Qualitative	**15**
Ethnography	2
Descriptive	10
Phenomenology	3
Quantitative	**76**
Cross-Sectional	48
Descriptive	3
Ecological	2
Interrupted Time Series	1
Longitudinal/prospective study	11
Regression discontinuity	1
Retrospective cohort	3
Surveillance	7
Mixed Methods	**7**
Population size	
0–100	16
100–1000	28
1000–10000	22
10,000 +	14
NR	18
Measures of police violence	
Datasets	**45**
Mapping Police Violence	9
The Counted	7
National Violent Data Reporting System	2
CDC WONDER	4
Fatal Encounters	11
Modified version of the Conflict Tactics Scale 2	1
National Vital Statistics System	1
Washington Post Fatal Force Database	5
National Trauma Data Bank	1
National Electronic Injury Surveillance System	1
Nationwide Inpatient Sample	1
Nationwide Emergency Department Sample	1
Police Initiative Database	1
Illinois Trauma Registry	1
Gun Violence Archive	1
Self-reported measures	**48**
Quantitative Single-Item Question	12
Quantitative Multi- Item Questions	24
Qualitative Single-Item Question	1
Qualitative Multi-Item Questions	9
Qualitative No Question but Theme Identified	8
Medical and Administrative Records	**13**
Death Certificates	3
Medical Records	3
Emergency Room Documentation	2
Complaints	4
Supervisor summaries	1

Note. Longitudinal designs included: longitudinal cohort (*n* = 11), interrupted time series (*n* = 1), retrospective cohort (*n* = 5), and regression discontinuity (*n* = 1). *CDC* Center for Disease Control for Prevention, *WONDER* Wide-Ranging Online Data for Epidemiologic Research. Since multiple measures were used in some studies, the total number of measures may be greater than 98

**Fig. 3:**
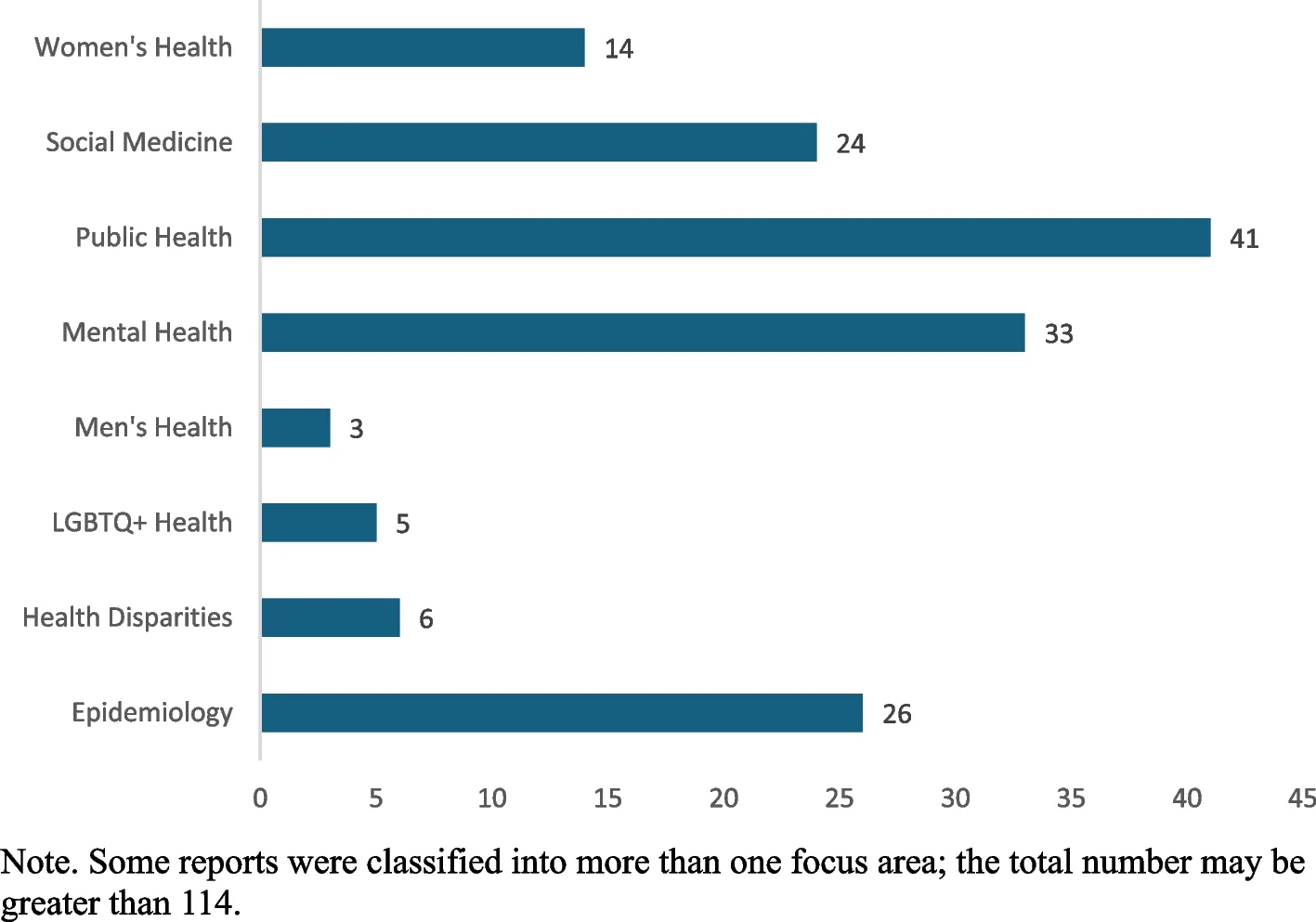
Health focus area of included reports

Figure 3 depicts the health focus areas and academic disciplines of the 114 included reports determined by the journal’s pertinent discipline. Some reports were identified as having more than one focus area. Forty-one reports were published in public health journals (e.g., *American Journal of Public Health*), 33 focused on mental health, 26 were specific to epidemiology, and 24 were in social medicine. While 14 reports were related to women’s health, few examined men’s health (*n* = 3) or lesbian, gay, bisexual, transgender and queer health (LGBTQ +) (*n* = 5). An additional six reports focused on health disparities. Included publications examined associations between police violence and risk factors for poor health outcomes, such as rates of sexually transmitted infections [22], preterm delivery [42], physiological stress [43], medical mistrust and unmet need for medical care [31], as well as emergency department utilization [44].

### Terms and definitions of police violence

We identified more than 100 terms for police violence in the health literature (Table 2), which we clustered into 22 categories, including abuse, assault, harassment, killing, and violence. The categories were determined based on verb usage in reviewed literature and account for synonyms, colloquial euphemisms, and other aggregations of terms used to describe the of police violence in the literature. “Police violence” was the most common term (*n* = 46), followed by “police brutality” (*n* = 12), “excessive use of force” (*n* = 11), and “police harassment” (*n* = 10). Only one report used the term “traumatic police stop.”

**Table 2:** Terminologies of police violence

Category	Terms for police violence	Frequency
Abuse (*n* = 18)	Police abuse	9
	Abusive or disrespectful experiences with police	1
	Abusive police encounters	1
	Abusive police interactions	1
	Abusive policing	1
	Physical abuse by law enforcement	1
	Physical abuse by police	1
	Physical and verbal abuse by police	1
	Physical brutality and abuse	1
	Police injuries	1
Aggression (*n* = 2)	Aggressive police behavior	1
	Aggressive policing practices	1
Assault (*n* = 5)	Police assault	2
	Assaulted, beaten	1
	Physical assault, theft, misconduct, humiliation	1
	Sexual assault by police	1
Beat (*n* = 3)	Assaulted, beaten	1
	Beaten by police	1
	Police beating	1
Brutality (*n* = 13)	Police brutality	12
	Physical brutality and abuse	1
Coercive (*n* = 2)	Police coercion	1
	Police sexual coercion	1
Deadly/Death (*n* = 19)	Police-involved death	3
	Police use of deadly force	3
	Law-enforcement related deaths	2
	Legal intervention deaths	2
	Mortality due to legal intervention	2
	Mortality due to legal intervention	2
	Death by legal intervention	1
	Civilian deaths by police	1
	Police use-of-force deaths	1
	Deadly force by police officers	1
	Police-related deaths	1
Discriminatory (*n* = 5)	Police discrimination	1
	Police torture, discrimination, threats, and public humiliation	1
	Profiling and harassment by police	1
	Police misconduct and microaggressions	1
	Police persecution	1
Disrespectful (*n* = 3)	Abusive or disrespectful experiences with police	1
	Disrespectful police behavior	1
	Egregious police behaviors	1
Fatal (*n* = 10)	Fatal police violence	3
	Fata police shootings	2
	Fatal interactions with police	1
	Fatal interactions from legal intervention	1
	Fatal encounters with police	1
	Fatal shootings of civilians by law enforcement officers	1
	Police use of fatal force	1
Force (*n* = 19)	Excessive use of force, excessive force, excessive police force	11
	Police use of force	3
	Less than lethal force by law enforcement, higher force, lower force	1
	Police use of fatal force	1
	Police use-of-force deaths	1
	Use of force	1
	Use of force incidents	1
Harassment (*n* = 13)	Police harassment	10
	Police harassment, misconduct, humiliation	1
	Police sexual harassment, threats and public humiliation	1
	Profiling and harassment by police	1
Hostile (*n* = 5)	Negative encounters with police	2
	Adverse police encounters	1
	Hostile police encounters	1
	Problems with the police	1
Illegal (*n* = 4)	Extrajudicial police actions	1
	Illegal policing practices	1
	Police extortion	1
	Procedural injustice or acts of police intrusiveness	1
Immoderate (*n* = 5)	Over policing	2
	Hyper policing	1
	Disproportionate nature of police use of force	1
	Police surveillance	1
Interactions or interventions (*n* = 12)	Legal intervention injuries	4
	Legal intervention	3
	Legal intervention deaths	2
	Police altercations	1
	Police interference	1
	Violent interactions with the police	1
Killing (*n* = 6)	Police killings	4
	Killed during legal interventions	1
	Exposure to police killings	1
Mishandling (*n* = 5)	Police misconduct	3
	Police misconduct and microaggressions	1
	Police mistreatment	1
Shooting (*n* = 5)	Fatal shootings of civilians by law enforcement officers	2
	Firearm injuries during legal interventions	1
	Officer-involved shootings	1
	Police shootings	1
Systemic (*n* = 3)	State violence	2
	Massive indiscriminative violent attacks	1
Trauma (*n* = 1)	Traumatic police stops	1
Violence (*n* = 56)	Police violence	46
	Fatal police violence	3
	Sexual violence from police	2
	Physical violence or psychological violence by police	2
	Detention violence	1
	Lethal police violence	1
	Violent policing	1

Note. Some reports used multiple terms to describe police violence; total number may be greater than 114

Conceptual definitions of these terms are presented in Table 3. The most common definition of police violence was police killings (*n* = 23). Four reports used the WHO classification of "police brutality." Other definitions reported in the literature included excessive use of force (*n* = 10) and experiencing physical, psychological, sexual violence, or neglect by police regardless of intent (*n* = 6). We identified three definitions for indirect police violence in the health literature:1) having a friend, relative, neighbor or colleague killed by police; 2) living in an area where fatal and non-fatal police violence occurred; and 3) worrying about oneself or a loved one being a victim of police violence. While all reports included a term for police violence, 38 reports did not include a definition.

**Table 3:** Conceptualizations of police violence

Terms for Police Violence	Conceptualization
Police abuse	• Ranges from prescribed exertion of violence on protesters to use of weapons in a way that is contrary to law enforcement's code of conduct, such as firing dispersion grenades directly at a protester. (Çelebi, E., et al., 2022) • Encompasses violent interactions, including but not limited to, sexual abuse by police, physical abuse by police, and verbal abuse by police that may or may have not resulted in injury, markings, or traumatic lesions. (Gilbert, L., et al., 2022; Iqbal, S., et al., 2021; Jackson, A.N., et al., 2020; Jun, H., et al., 2018; Lorin de la Grandmaison, G., et al., 2007) • An affirmative response to any of the following items: two items for unjustified arrests (been arrested or detained for carrying a syringe; been arrested or detained after the police 'planted' syringes or drugs on you); one item for beatings by officers, two items for sexual violence (been forced to have sex with a police officer; been forced by a police officer to have sex with other people), and three other items (had syringes taken from them by the police, been forced to give money to the police to keep from being arrested, had their driver's license/registration/passport taken from them by a police officer). (Schoenberger, S. F., et al., 2022) • The words assaulted, beaten, excessive force, and police brutality as quoted by the patient, an intent to pursue legal action, a complaint of inappropriate force, or reference by the patient to force that is not within common police guidelines (e.g., strangulation). (Strote, J., & Hickman, M. J., 2018) • Other definitions not specified: Davis, S., et al., 2009, Greene, C., et al., 2022, Hayashi, K., et al., 2013, Novich, M., & Hunt, G., 2017
Police brutality	• Experiences and appraisal of negative encounters with the police; it further encompasses unreasonable use of physical force, as well as actions by police meant to intimidate, harass, or dehumanize. (Alang, S., et al., 2020) • Police (in)action that dehumanizes, regardless of conscious intent, and it encompasses psychological intimidation verbal abuse, and physical assault. It includes emotional and physical abuse, verbal assault, psychological intimidation, physical and sexual violence, and neglect. (Alang, S., et al., 2021^b^; Alang, S., et al., 2022) • A form of structural violence that is socially arranged, routinized, normalizes state-sanctioned anti-Black violence, and even deems this violence and anti-Black surveillance as necessary to preserve 'collective' safety. (Alang, S., et al., 2022) • An anticipatory stressor; Anticipatory stressors are significant ongoing concerns about events or experiences that may occur in the future. (Alang, S., et al., 2022) • As defined by the WHO which classifies police brutality as a form of violence and defines violence itself as *'the intentional use of physical force or power, threatened or actual, against oneself, another person, or against a group or community that either results in or has a high likelihood of resulting in injury, death, psychological harm, maldevelopment or deprivation*' [13]; Fedina, L., et al., 2018) • Other definitions not specified: Alang, S., et al., 2021^a^, Çelebi, E., et al., 2022, Friedman, J., et al., 2021, Leun, Z., et al., 2022, Mehra, R., et al., 2022, Novich, M., & Hunt, G., 2017
Police-related death	• Those killed by law enforcement, homicide, committed by law enforcement, death while victim in custody. (Arseniev-Koehler, A., et al., 2021) • Occurrences of people who have been killed through interactions with law enforcement. (Browning, C., et al., 2021; Feldman, J.M., et al., 2017) • Death caused by law enforcement officials other than legal execution. (Drowos, J., et al., 2015) • Cause of death was identified as asphyxiation, beating, a chemical agent, a medical emergency, a Taser, or a gunshot. (Edwards, F., et al., 2018; Feldman, J.M., et al., 2017) • Any deaths arising directly from encounters with law enforcement, including but not limited to, people who were shot, tasered, and struck by police vehicles as well as those who died in police custody. (Feldman, J.M., et al., 2017) • Perceptions of threatening and distressing actions by the state; fatal police violence can therefore be understood as a population-level stressor with potential psychosocial and physiologic implications. (Jahn, J., et al., 2021) • Incidents that occurred in New York state during 2013–2016, involved an unarmed Black victim and were not a murder-suicide committed by the police officer. (Liu, S., Lim, S., & Gould, L., 2021) • Fatalities in police custody or involving the police that would not have occurred in the absence of police intervention. (Schwartz, G.L., & Jahn, J.L., 2022) • ICD-9: “… homicides” was defined as ICD-9 E (external cause of injury) codes E960 through E977. The ICD-9 definition of legal intervention (E970-E977) includes, *'injuries inflicted by the police or other law-enforcing agents... in the course of arresting or attempting to arrest lawbreakers, suppressing disturbances, maintaining order, and other legal action*.' (Sikora, A.G., & Mulvihill, M., 2002) • When a person dies as a result of being chased, beaten, arrested, restrained, shot, pepper sprayed, tasered, or otherwise harmed by police officers, whether on-duty or off-duty, intentional or accidental. (Smith Lee, J., & Robinson, M., 2019) • A lethal police encounter is a case where a person dies due to being beaten, shot, denied medical attention, or otherwise harmed by a member of law enforcement. (Talbert, R., 2022) • *Exposure to Police Killings* include (1) total police killings of Black people and (2) unarmed police killings of Black people. For a resident to have been exposed to a police killing, the event must meet two conditions: (1) it occurred in the 365 days before one's BRFSS survey interview date, and (2) it occurred in one's state of residence. (Talbert, R., 2022) • ICD-10: Death due to injuries inflicted by law enforcement. (Ruch, D.A., et al., 2021) • Other definitions not specified: Feldman, J.M., et al., 2017, Kleck, G., 2007, Liasidis, P.K., et al., 2021, Miller, T., et al., 2017; Mitchell, J., & Chihaya, G.K., 2022, Schwartz, G.L., & Jahn, J.L., 2020, Sheppard, K.G., & Stowell, J.I., 2022
Police harassment	• Intersectional structural vulnerability framework. (Friedman, J., et al., 2021) • Aggressive, proactive policing leading to victimization. (Greene, C., et al., 2022) • Experiences of discrimination and race-related events. (Kalinowski J., et al., 2022) • Other definitions not specified: Feelemyer, J., et al., 2021, Friedman, J., et al., 2021, Wendel, M., et al., 2022, West, B. et al., 2020
Hyper-policing	• An aggressive form of policing involving intense police surveillance and designation of neighborhoods and their residents as potential or actual criminals. (Feelemyer, J., et al., 2021) • Other definitions not specified: Domínguez, D., et al., 2022, Mehra, R., et al., 2022; Wendel, M., et al., 2022
Police-related injury	• ICD-9: Legal intervention (those caused by law enforcement), as per the International Classification of Diseases, 9th Revision (ICD-9); Patients injured because of contact with law enforcement personnel were identified using the ICD-9 external cause of injury codes (ECODES) ranging from E970 to E977, which are defined as legal intervention cases. (Feldman, J.M., et al., 2016; Holloway-Beth, A., et al., 2019; Mooney, A.C., et al., 2018) • ICD-10: For decedents killed by law enforcement, mortality records are assigned a diagnostic code, under the International Classification of Diseases, 10th Revision (ICD-10; Geneva, Switzerland: World Health Organization; 1992), indicating legal intervention, defined as injuries inflicted by the police or other law- enforcing agents, including military on duty, while arresting or attempting to arrest lawbreakers, suppressing disturbances, maintaining order, and other legal action. (Feldman, J.M., et al., 2017; Liasidis, P.K., et al., 2021; Ormseth, C.H., et al., 2022) • Injuries inflicted by the police or other law-enforcing agents, including military on duty, while arresting or attempting to arrest lawbreakers, suppressing disturbances, maintaining order, and other legal action. (Holloway-Beth, A., et al., 2016) • Other definitions not specified: Miller, T., et al., 2017
Police misconduct	• Abusive interactions outside the scope of legal enforcement practices, such as harassment, sexual assault, damage of personal property, physical violence, or coerced sex in exchange for no arrest. (Fehrenbacher, A., et al., 2020) • Extrajudicial police actions and/or unauthorized confiscation of unused syringes, routine harassment, or physical or sexual violence. (Friedman, J., et al., 2021) • Police discriminatory and unlawful behavior, ranging from microaggressions and verbal abuse to sexual misconduct and physical violence. (Girardi, R., 2022) • Evidence planting by police (in the form of police placing illicit drugs on an individual) as an indicator of direct encounters with police. (Hayashi, K., et al., 2013) • Procedural injustice or acts of police intrusiveness, such as harsh language, threats of force, or use of force. (Jackson, D.B., et al., 2021) • Other definitions not specified: Borrero, M., 2001, Friedman, J., et al., 2021
Negative interaction with police	• Negative encounters with police refer to a social determinant of health that shapes how people live, work, and play; also, refers to police (in)action that dehumanizes, even in the absence of conscious intent, and includes verbal assault, psychological intimidation, and physical, sexual, and emotional violence. (Alang, S., et al., 2021) • Concerns about disrespectful treatment were typically identified as disproportional or inappropriate use of force during a stop [45]. Perceptions of disrespect occurred both in the context of stops deemed legitimate (i.e. police encounter because of criminal behavior) and those deemed illegitimate (i.e. police encounter not perceived as justified). (Novich, M., & Hunt, G., 2017) • Other definitions not specified: Borrero, M., 2001, Freedman, A., et al., 2022, Jackson, D.B., et al., 2021
Police excessive use of force	• Exposure and other common uses of force descriptors include unnecessary physical contact and unnecessary display of a weapon. (Freedman, A., et al., 2022) • Low levels of force, such as grabbing, pushing or shoving. (Holloway-Beth, A., et al., 2019) • Type of force used was differentiated into higher force and lower force. The high force was defined as a canine bite, use of a baton, flash-bang device, strike, use of hands, fists, or feet, kick, taser, chemical sprays, pepper balls, or bean bag rounds. The lower force was everything else noted and included joint pressure, pushes, take-downs, and handcuffing. (Lewis, S., & Bueno de Mesquita, B., 2020) • The application of force greater than that which a 'reasonable' and 'prudent' law enforcement officer would use under the circumstances. (Motley, R.O., Jr, & Joe, S., 2018) • Direct (e.g., victimization or witnessing the event in person) or indirect (e.g., learning about the event from someone or through media) exposure to intentional acts of interpersonal violence (e.g., assaults, homicide, and other types of serious physical harm) committed in public settings by individuals who are not intimately related to the victim. (Motley, R.O., Jr, et al., 2022) • Experiencing, witnessing, or learning about the police forcefully stopping someone via 'throwing, slamming, or grabbing individuals as well as shoving, beating or handling people roughly'. (Novich, M., & Hunt, G., 2017) • Defined as levels of force by the police office and level of resistance by the suspect such that if the maximum level of officer force was higher ("force") then the force factor would be equal to force-resistance indicating that the officer used 1 level of force higher than the degree of suspect resistance. (Strote, J., & Hickman, M. J., 2020) • Other definitions not specified: Borrero, M., 2001, Çelebi, E., et al., 2022, Feldman, J.M., et al., 2016, Leun, Z., et al., 2022, Mooney, A.C., et al., 2018
Police violence	• The intentional and unintentional conduct of police officers that dehumanizes people, and it includes the perpetration of psychological violence and intimidation, neglect, verbal abuse, and physical and sexual violence. (Alang, S., et al., 2021) • Having experienced episodes of violence (physical, verbal, psychological, or sexual) by police or security forces. (Aristegui, L., et al., 2022) • Having been arrested or beaten up by police in the last six months. (Bhattacharjee, P., et al., 2019) • Being physically assaulted or arrested by law enforcement during sex work. (Bhattacharjee, P., et al., 2020) • Years of life lost due to encounters with law enforcement. (Bui, A., et al., 2018) • Police killings in each state. (Burch, A., & Jacobs, M., 2022) • Occurs when law enforcement agents respond with the use of force in either a proportionate or a disproportionate way during protests. (Çelebi, E., et al., 2022) • Police physical violence was defined as being hit, punched, slapped, or otherwise physically hurt or threatened or hurt with a weapon by clients/intimate partners/police. (Decker, M.R., et al., 2021) • Physical and verbal violence from police. (Lacombe-Duncan, A., & Olawale, R., 2022) • Police sexual violence additionally included police extortion in the form of pressured sex in exchange for leniency as a form of sexual coercion unique to this and other criminalized populations. (Decker, M.R., et al., 2021) • WHO domains of violence [13]; *'the intentional use of physical force or power, threatened or actual, against oneself, another person, or against a group or community that either results in or has a high likelihood of resulting in injury, death, psychological harm, maldevelopment or deprivation*'. (DeVylder, J., et al., 2017; DeVylder, J., et al., 2018; Fedina, L., et al., 2018) • Violence embedded in a social structure that perpetuates fear and terror, internalized stigma, and a sense of helplessness and fatality especially among women (Lunze, K., et al., 2016) • Including extortion, coercion, discrimination, and physical, sexual, and psychological violence. (Mukherjee, T., et al., 2022; West, B. et al., 2020) • An affirmative to any of the following: Witnessed or heard close-up gunfights between police and bandits; Had a relative, friend, neighbor, or colleague of yours been killed by police?; You have been verbally insulted or humiliated by a police officer(s); Was physically assaulted by a police officer(s). (Pexioto, E.M., et al., 2022) • Systematic police persecution to remove the children from the streets against their will; actions that had the deliberate intent to humiliate them with verbal or physical aggression; and through alleged sexual abuse, revealed by the children in a veiled manner. (Ribeiro, M.O., 2008) • Other definitions not specified: Aciksoz, S., 2016, Alang, S., et al., 2022, Browning, C., et al., 2021, Davis, S., et al., 2009, Domínguez, D., et al., 2022, Edwards, F., et al., 2018, Fehrenbacher, A., et al., 2020, Feldman, J.M., et al., 2016, Freedman, A., et al., 2022, Friedman, J., et al., 2021, Gilbert, L., et al., 2022; Greene, C., et al., 2022, Jackson, D.B., et al., 2021; Jun, H., et al., 2018; Mitchell, J., & Chihaya, G.K., 2022; Salas-Hernández, L., et al., 2022; Smith Lee, J., & Robinson, M., 2019; Wendel, M., et al., 2022; Young, A., et al., 2007, Zare, H., et al., 2022
State violence	• State challenges to medical humanitarianism and neutrality, atmospheric violence, and the state’s deployment of riot control agents (often generically referred to as teargas) against civilian populations which manifests as police violence. (Aciksoz, S., 2016) • Any aspect of culture (e.g., narratives) that is, used to justify either direct or structural violence. (Domínguez, D., et al., 2022) • A social structure that produces, maintains, and amplifies inequitable life chances. (Domínguez, D., et al., 2022) • Involves *'somatic incapacitation, or deprivation of health, alone, at the hands of an actor who intends this to be the consequence*'. (Domínguez, D., et al., 2022) • Direct and structural violence enacted by police officers, with structural violence meaning "*'social arrangements that put individuals and populations in harm's way'*". (Wendel, M., et al., 2022) • Other definitions not specified: Lacombe-Duncan, A., & Olawale, R., 2022

Note. ^a, b^Represent distinct publications by the same authors in 2021; alphabetical suffixes are used to differentiate them. Not all reports (*n* = 38) provided a definition of police violence; total may be less than 98

### Measures of police violence

Several measures of police violence were identified in this review. The three most common measures were counts of fatal police encounters using datasets (e.g., *Mapping Police Violence, The Counted, Fatal Encounters*), self-report, and medical chart reviews (Table 1). Measures of police violence are summarized below. Full descriptions of the databases and measures can be found in Table 4.

**Table 4:** Operationalizations of police violence: key databases and measures

Databases	Description
California's Ursus Program Dataset	This compiles data on all citizen complaints of excessive police force in California. The dataset includes data at municipality, county and state levels
Centers for Disease Control and Prevention' Wide-Ranging Online Data for Epidemiologic Research (WONDER)	Freely accessible website for accessing public health information
Centers for Disease Control and Preventions Compressed Mortality File (CMF)	The CMF is public domain mortality and population database with annual national, regional, state, and county-level information on the underlying cause of death as stated on the death certificate according to age, gender, race, and cause, for all legal residents
Fatal Encounters Database	Fatal Encounters is a non-partisan, comprehensive database that synthesizes public information about individuals in the United States killed during interactions with police since January 1, 2000
Gun Violence Archive	The GVA is a not-for-profit corporation formed in 2013 to provide a near-real-time online archive of firearm violence incidents (both fatal and nonfatal) in all 50 states and the District of Columbia. Data are collected from more than 2500 media, law enforcement, government, and commercial sources daily. The GVA codes and labels incidents with broad categories (e.g., suicide, homicide) including officer-involved shootings. The GVA provided us with incident-level data on all officer-involved shootings in which the suspect or perpetrator was shot and killed (including incidents in which the victim died from a self-inflicted gunshot wound) for all 50 states in 2015
Illinois Trauma Registry	The ITR receives data from all level 1 and 2 trauma units in Illinois. Data are entered into a standardized electronic form at each respective trauma center by trained personnel from medical records upon discharge of a patient. The trauma centers are required to report all patients (1) sustaining traumatic injuries (ICD-9-CM external injury codes E800-995) and admitted to a trauma center for > 12 h, (2) transferred to a level I or II center or (3) are dead-on-arrival (DOA) or die in the emergency department. The data found in the ITR contains demographics information such as the age, gender, and the ethnic/racial background of the patient. In addition, the database includes the mechanism of injury, which is used as the exposure variable, in addition to health indicators and patient outcomes such as the patient diagnoses, measures of injury severity, and hospital procedures, total hospital charges and payer source
Invisible Institutes Citizens Police Data Project	Formal complaints filed against Chicago police through June 2018
International Classification of Disease (ICD-9 and ICD-10)	ICD-9 codes (E970-E97, 994.8) and ICD-10 code for external-cause-of-injury (Y35.0 to Y35.4 or Y35.6 to Y35.7), which includes deaths resulting from ‘injuries inflicted by the police or other law-enforcing agents, including military on duty, in the course of arresting or attempting to arrest lawbreakers, suppressing disturbances, maintaining order, and other legal action’. This included legal interventions involving firearm discharge (Y35.0), explosives (Y35.1), gas (Y35.2), blunt objects (Y35.3), sharp objects (Y35.4), other specified means (Y35.6), or unspecified means (Y35.7) and excluded deaths involving legal execution (Y35.5)
Mapping Police Violence (MPV) Database	MPV verifies data quality and completeness through verification by media outlets, obituaries, criminal records databases, and police reports and, when possible, includes additional information such as race, vehicular use, and armament status. Compared to other sources of police killings, such as the Washington Post Fatal Encounters database, MPV includes deaths that result from chokeholds, batons, tasers, or other means
National Trauma Data Bank	Maintained by the American College of Surgeons' Committee on Trauma. The NTDB is the largest trauma data repository in the world with voluntary participation of trauma centers across the United States. The data contained in the NTDB are standardized at the time of submission using the validation system and rules defined in the National Trauma Data Standard data dictionary
National Violent Death Reporting System (NVDRS)	NVDRS is the only state-based surveillance reporting system that pools more than 600 unique data elements from multiple sources into an anonymous database. NVDRS covers all types of violent deaths 'including homicides and suicides' in all settings for all age groups. https://www.cdc.gov/violenceprevention/datasources/nvdrs/index.html
National Vital Statistics System (NVSS)	The National Vital Statistics System (NVSS), administered by the US government and based on state death certificates, is the longest-running national data source on law-enforcement-related deaths (i.e., those involving fatal injuries inflicted by law enforcement). The NVSS has identified law-enforcement-related deaths since 1949, following the inclusion of 'injury by intervention of police' as a diagnostic category in the 6th revision to the International Classification of Diseases (ICD). While the category has since been renamed as 'legal intervention' its definition remains unchanged up to the current ICD revision, ICD-10: 'injuries inflicted by the police or other law-enforcing agents, including military on duty, in the course of arresting or attempting to arrest lawbreakers, suppressing disturbances, maintaining order, and other legal action'. A designation of legal intervention does not depend on whether the use of force resulting in the injury was lawful or whether the injuries were inflicted intentionally
Police Data Initiative	https://www.policedatainitiative.org/datasets/use-of-force/. From that resource, only two (Indianapolis, Indiana, and Wichita, Kansas) of thirty published police incident data sets included sufficient injury and hospitalization information to make it possible to test the outcomes of police encounters as a function of race while approximating a natural experiment
The Guardians online database (The Counted, 2016)	People killed by police reported to The Guardian. https://www.theguardian.com/us-news/series/counted-us-police-killings
The Washington Post Fatal Force Database	The Washington Post has been compiling a database of every fatal shooting in the US by a police officer in the line of duty by culling local news reports, law enforcement websites and social media, and by monitoring independent databases such as KilledbyPolice.net and Fatal Encounters. Compared to other open-source datasets, the Washington Post's database applies a more restrictive case definition and only includes shootings in which a police officer, in the line of duty, shoots and kills a civilian. It does not track deaths of people in police custody, fatal shootings by off-duty officers or non-firearm deaths (Tate et al., 2016)
Published Surveys, Measures, and Questionnaires	**Description**
Police Practice Inventory (PPI)	This 27-item measure assesses police violence across the four WHO domains of violence: physical, psychological, sexual, and neglectful (Cooper et al., 2004) Cooper, H., Moore, L., Gruskin, S., & Krieger, N. (2004). Characterizing perceived police violence: Implications for public health. *American Journal of Public Health*, 94(7), 1109–1118. https://doi.org/10.2105/AJPH.94.7.1109
Index of Race-Related Stress Brief (IRRS-B)	The IRRS-B is a 22-item assessment tool specifically designed to evaluate the stress that African Americans experience due to encounters with racism. This instrument measures four key constructs: individual racism, collective racism, institutional racism, and cultural racism. Items on police interactions were included Seaton, E. K. (2003, 2003/08/01). An Examination of the Factor Structure of the Index of Race-Related Stress among a Sample of African American Adolescents. *Journal of Black Psychology, 29*(3), 292–307. https://doi.org/10.1177/0095798403254211
Experiences of Discrimination Scale	This nine-item measure about lifetime experiences of racial discrimination includes one item about discrimination by police or in the courts Krieger N, Smith K, Naishadham D, Hartman C, Barbeau EM. Experiences of discrimination: validity and reliability of a self-report measure for population health research on racism and health. *Soc Sci Med.* 2005;61(7):1576–1596. 10.1016/j.socscimed.2005.03.006
Modified Classes of Racism Frequency of Racial Experiences (M-CRFRE)	M-CRFRE includes 16 items capturing exposure to racism-based police violence across three domains: victim, witness in person, and seen in the media Motley RO, Joe S, McQueen A, Clifton M, Carlton-Brown D. Development, construct validity, and measurement invariance of the Modified Classes of Racism Frequency of Racial Experiences Measure (M-CRFRE) to capture direct and indirect exposure to perceived racism-based police use of force for Black emerging adults. *Cultur Divers Ethnic Minor Psychol*. 2023;29(4):575–589. https://doi.org/10.1037/cdp0000525
Detroit Area Study Discrimination Questionnaire	28-item measure that reports discrimination based on three distinct categories: frequency of everyday mistreatment, recent episodes of discrimination (within the last 12 months), and reports of lifetime history of discrimination. Items related to police violence include, “have you ever been unfairly stopped, searched, questioned, physically threatened, or abused by the police?” Taylor TR, Kamarck TW, Shiffman S. Validation of the Detroit Area Study Discrimination Scale in a community sample of older African American adults: the Pittsburgh healthy heart project. Int J Behav Med. 2004;11(2):88–94. https://doi.org/10.1207/s15327558ijbm1102_4
JuviPool questionnaire	The Core JVQ includes 34 items that cover the following domains: conventional crime, child maltreatment, peer & sibling victimization, sexual victimization, and witnessing & indirect victimization Hamby, S., Finkelhor, D., Turner, H., & Kracke, K. (2011). The Juvenile Victimization Questionnaire toolkit. Retrieved from https://www.unh.edu/ccrc/juvenile-victimization-questionnaire
Perceptions of Police Scale	Asks participants the degree to which they agree with twelve statements that measure an individual’s perceptions of police. Sample items include, “police protect me”; “police treat people fairly”; and “police do not discriminate.” Nadal, Kevin & Davidoff, Kristin. (2015). Perceptions of Police Scale (POPS): Measuring Attitudes towards Law Enforcement and Beliefs about Police Bias. Journal of Psychology and Behavioral Science. 3. 10.15640/jpbs.v3n2a1
Survey of the Health of Urban Resident	Respondents were presented with examples of police actions: cursing at them, searching, frisking, threatening arrest, handcuffing, threatening with a ticket, shoving or grabbing, hitting or kicking, using pepper spray or other chemicals, using electroshock weapons like stun guns, and pointing a gun. They were asked whether these actions had never happened, occurred once or twice, a few times a year, monthly, or weekly Alang, S., Pando, C., McClain, M., Batts, H., Letcher, A., Hager, J., Person, T., Shaw, A., Blake, K., & Matthews-Alvarado, K. (2021). Survey of the Health of Urban Residents: a Community-Driven Assessment of Conditions Salient to the Health of Historically Excluded Populations in the USA. *Journal of racial and ethnic health disparities*, *8*(4), 953–972. https://doi.org/10.1007/s40615-020-00852-1

Note. This table provides key databases and measures used to assess police violence. We specifically list those databases and measures investigators can easily access for future research studies

#### Datasets

Forty-five of the 98 included studies used datasets to measure police violence. Two such datasets are *Mapping Police Violence *[46] and *The Counted* [47], which use crowdsourced materials from regional news outlets, research groups, and other open-source reporting projects to collect information on police-related fatal encounters. The Centers for Disease Control and Prevention (CDC) maintains the National Violent Death Report System (NVDRS), a comprehensive governmental database of all deaths in participating states that result from homicide, suicide, legal intervention (excluding legal execution), unintentional shooting, and injuries of undetermined intent [48]. NVDRS uses information obtained from death certificates, medical examiner/coroner reports, and law enforcement reports where the abstractors at the state level code the cause of death as a "legal intervention." Only one study used the *Gun Violence Archive* [49] *(GVA),* a near-real-time online archive of firearm violence incidents (both fatal and nonfatal) in all 50 states and the District of Columbia. Of the publicly available data sets used, *The Washington Post* had the most restrictive case definition, "only including shootings in which a police officer, in the line of duty, fatally shoots a civilian. It does not track deaths of people in police custody, fatal shootings by off-duty officers, or non-firearm deaths" [50]. Some studies combined multiple datasets (*n* = 7). One such study [51] cross-linked individual-level mortality data from the 2015 NVDRS and five other open-source datasets: Fatal Encounters, Mapping Police Violence, The Guardian's "The Counted," Gun Violence Archive, and The Washington's Post's "Fatal Force Database.”

#### Self-report

Forty-eight studies measured police violence by self-report (Table 1). In quantitative studies, common items included “have you ever had negative encounters with the police?”; and “has a police officer slapped, punched, shoved, kicked, shaken or otherwise physically hurt you?” One study [52] used a modified version of the Classes of Racism Frequency of Racial Experiences Measure (M-CRFRE) [53] to assess perceived racism-based police violence across three domains (victim, witness in person, seen in media). Qualitative interview guides assessed experiences or perceptions of police violence among at-risk and historically marginalized populations such as people who inject drugs, people living with mental illnesses, African American and Black men, women and children, and the intersectionality of race, gender, or sexual identity with police violence experiences. Participants described their experiences in detail as " ‘we were hanging out on the street when police approached us swearing. They hit me, threw me against the ground, put a gun in my face, threw me against the wall…’” [54]. Another participant described their experience as “‘I've been propositioned by police, I've traded my freedom and drugs from police for sex.’” [55].

Three studies in this review used the Police Practices Inventory (PPI) [28, 56, 57]. The PPI is a 27-item measure developed based on the four WHO domains of violence (i.e., physical, sexual, psychological, and neglect) [48]. The PPI consists of 5 dichotomous items: 1) “has a police officer ever used a gun, baton, taser, or other weapon on you?”; 2) “has a police officer ever hit, punched, kicked, dragged, beat, or otherwise used physical force against you?”; 3) “has a police officer ever engaged in nonphysical aggression toward you, including threatening, intimidating, stopping you without probable cause, or using slurs?”; 4) “has a police officer ever forced inappropriate sexual contact on you, including while conducting a body search in a public place?”; and 5) "have you ever called or summoned the police for assistance and the police either did not respond, responded too late, or responded inappropriately?” Respondents were then asked to report how many times in the past 12 months they were exposed to each experience. Notably, the PPI was the only self-report measure identified in this scoping review with psychometric data. The PPI was found to have moderate to strong test–retest reliability for each WHO domain of police violence: physical (*K* = 0.58), sexual (*K* = 0.40), psychological (*K* = 0.60), and neglectful victimization (*K* = 0.64) [58].

### Medical records and administrative records

Six studies in this review used electronic medical records, death certificates, and medical examiner/coroner reports to assess police violence with International Classification of Diseases (ICD) codes [42, 59–63]. Two studies evaluated injuries in the emergency department as a result of legal interventions [62, 63]. ICD-9 and ICD-10 codes (i.e., Y35.0-Y35.9) were used to identify any injury sustained because of an encounter with any law enforcement official, serving in any capacity at the time of the encounter. Death certificates and medical examiner reports were reviewed to monitor deaths while in police custody. Four studies reviewed complaints of excessive force by police documented in police department records, emergency room documentation [64–67], and one study used the incidence reports from the California Ursus Program Dataset [68].

## Discussion

This scoping review aimed to map existing definitions and measurement approaches to police violence in the health literature. Police violence has received relatively new attention in public health and health services research, as evidenced by the growing number of publications identified in this review after 2020 (Fig. 2). This coincides with the murder of George Floyd by Minneapolis police officer Derek Chauvin and the COVID-19 pandemic which highlighted social inequities and health disparities [69–71]. In the health literature, most of this work is being conducted in public health, epidemiology, and social medicine. While emerging research suggests that police violence is associated with premature death [72], increased cardiovascular disease (CVD) risk [60], depression, anxiety [73], and posttraumatic stress disorder [74], there is a lack of clarity on how to define and measure the phenomenon. We identified more than 100 terms for police violence, varying from "police harassment” to “police assault,” and “death by legal intervention.” One inconsistency in the definitions noted in this review was whether physical violence (fatal, non-fatal, sexual, or non-sexual) was the defining characteristic of police violence. While few studies, considered any act by police that dehumanizes people (e.g., intimidation, neglect, verbal abuse) a form of police violence [22], other studies [43, 75, 76] focused on deaths during interactions with the police. In addition, several studies [52, 77–79] included the intention of the police in their definition of police violence. However, Alang et al. (2021), and Smith (2019), do not include police intent when determining if the use of force was excessive. These discrepancies make it difficult to rigorously assess the impact of police violence. The WHO definition of violence provides a useful reference point because it incorporates multiple domains of harm (e.g., physical, psychological, sexual, and neglectful) that are relevant to understanding police encounters. Not accounting for multiple domains of harm can lead to the underreporting of non-physical forms of police violence. Narrow conceptualizations may overlook documented structural and discriminatory policing practices such as racial profiling that disproportionately affect minority communities and can produce psychological harm.

Furthermore, available data suggest that current measures do not fully capture the pervasiveness of police violence. Open-source, governmental, and publicly available datasets are a common measure used by researchers to study the risk factors of police violence [33, 80, 81], ways to reduce incidents of police violence [45, 54], and the prevalence of police violence [34, 35, 82, 83]. However, a recent study found that more than half of police-related deaths between 1980 and 2019 were misclassified or underreported in the US National Vital Statistics System (NVSS) [84], and evidence suggests that more than half of police violence incidents were not counted using combined non-governmental and open-source databases [85]. Since definitions of police violence may differ across databases, pertinent interactions and subsequent documentation may vary. This review highlights that these databases are inconsistently applied in the health literature, which may perpetuate inaccurate estimates. While nine studies used Mapping Police Violence and six studies used *The Counted*, others combined multiple sources, resulting in varying estimates of police violence.

This review found that self-reporting is a common method for measuring non-fatal police violence within the health literature. This approach was widely utilized both in the U.S. and internationally [86–89]. Self-report was common across all research designs, but the questions used to elicit the information varied widely. Single-item measures, such as “Have you ever been beaten by police?”, may not fully reflect experiences of police violence. Multiple-item scales assess police violence more broadly, possibly including the varying domains of violence. To limit the inconsistency of measures in the literature, several novel and validated self-report measures have been developed including the PPI [48]. Consistent definitions and standardized measures are essential to improve comparability across studies and advance understanding of health impacts.

While our study did not look at perceptions of police violence, future studies should examine how anticipatory stress related to police violence affects health, particularly among disadvantaged and vulnerable populations. Anticipatory stress related to police violence has been associated with psychological distress and suicide attempts among adults aged 18 and older from diverse U.S. community samples [28, 29] and with depressed mood among women [90]. Police involved in use-of-force incidents also face significant health risks, including acute stress responses and adrenaline dumps that lead to sleep disruption and fatigue [91]. Repeated exposure to high-intensity encounters increases vulnerability to PTSD and physical injury, with these cumulative health challenges extending beyond individual well-being to affect organizational stability through decreased morale and lower retention rates among officers [92, 93]. Similar to efforts to generate consistent measures of racism in the health literature [94], there is a need for *shared understanding and language to advance equity and justice.*

The definitions and measures described in this review highlight important concerns and challenges for the state of investigating police violence and its health impacts. The absence of consistent terminology, a reliable operationalization, and an explicitly stated definition of police violence that captures the various forms of police violence underpins the fragmentation in measuring this detrimental phenomenon. It is important to note that this issue has been raised previously by multiple reviews concerning the state of police violence exposure measurement [7, 34, 35, 51]. However, this is the first review to focus on terms, definitions, and measures in the health literature.

### Limitations and strengths

There are several limitations and strengths to the current scoping review. We used a scoping review methodology and considered broad definitions of police violence, which provides a comprehensive mapping of the literature but also introduces limitations. Broader conceptualizations may reduce comparability across studies that use narrower, force-only definitions. We limited our search to English language publications and only included journal articles within our chosen health databases to align with our research aims, which may exclude additional reports consisting of a term and/or measure of police violence. Applying WHO’s definition to police violence requires careful consideration of how ‘power’ is exercised beyond physical force. While this inclusive approach captures structural and psychological harms, it also raises challenges for distinguishing misconduct from routine policing. Future research should aim to develop operational definitions that balance conceptual breadth with practical clarity, ideally through cross-disciplinary consensus. Since this is a scoping review, we did not include risk of bias or assess methodological quality of the evidence. A limitation in the databases we searched is the lack of a controlled vocabulary for police violence; for example, there is no Medical Subject Heading (MeSH) for police violence.

Although police violence is ill-defined in the health literature, a key strength of this review is our comprehensive search strategy to ensure a broad capture of both MeSH and non-MeSH reports [39]. An additional strength of this review is the use of a scoping review methodology, which allows for a comprehensive mapping of existing literature and accommodates diverse study designs and sources. Finally, this is the first scoping review to describe and synthesize definitions and measures of police violence in the health literature, which is necessary to identify gaps in the literature and opportunities for future areas of scholarship.

### Implications for practice, policy, and research

#### Practice

By incorporating terms and measures for police violence, healthcare providers can better identify this social determinant of health during assessments, document it consistently, and connect patients to appropriate support and resources [95]. This review found that medical records offer an innovative approach to measuring police use of force, but they often underrepresent the full spectrum of police-related injuries (especially less severe or fatal cases), and lack contextual details, as noted by prior research [96]. Our scoping review highlights inconsistent use of terms related to police violence in the literature, which contributes to these gaps. One way to address this is by training health care professionals to screen for a history of police-related trauma (e.g., abrasions from too tight handcuffs, physical injuries from excessive use of force, inappropriate sexual contact during police encounters) during clinical visits. Clinicians must assess, document, and report police related injuries and trauma to better identify patients’ health and social needs [97]. A systematic approach to data collection of police violence in healthcare settings is needed.

### Policy

Police violence is a complex issue influenced by various factors, including policy, environmental conditions, and systemic elements [3, 98, 99]. Our scoping review highlights how standardized language is necessary for comprehensive data collection, which in turn can influence policy aimed to mitigate excessive use of force, defined as more force than warranted in a particular situation [100] leading to fatal and nonfatal police violence. Our review found wide variation in terminology and measurement tools across studies, which limits comparability and obscures the true prevalence of fatal and nonfatal police violence. Addressing these gaps through shared definitions and validated measures can strengthen accountability frameworks and inform legislation such as the George Floyd Justice in Policing Act of 2021 [101], which seeks to improve data transparency, restrict qualified immunity, and establish national use-of-force standards. Although this bill faces significant legislative challenges, several states—including New York, Colorado, New Mexico, New Jersey, California, and Arizona—have amended qualified immunity [102–105]. Future research should examine whether improved definitional clarity and standardized measurement contribute to more effective policy reforms and reductions in police violence.

### Research

Individuals and communities who suffer the most (e.g., Black Americans, LGBTQ +, people living with mental illnesses) from the multifaceted impacts of police violence are underrepresented in research, and similar patterns are observed among marginalized groups globally [106]. Although disparities in police violence are well-documented, only a handful of studies have examined the meaning and lived experiences of police violence among these communities [107–111]. One such study included in this review is the U.S Transgender Survey, the largest survey examining the experiences of transgender people in the U.S., which reported high levels of mistreatment and harassment by the police [112]. Beyond the U.S., research such as Miles-Johnson’s study in Australia demonstrates that transgender individuals globally experience police mistreatment and harassment, reinforcing the need for cross-national strategies to address these systemic issues [113]. As noted in Fig. 3, our review suggests there is a growing body of research examining the associations between police use of violence and mental health outcomes, yet studies on police use of violence among people living with mental illness is lacking. In addition, there is scant literature exploring the mechanism by which police violence impacts health and contributes to health inequities. Further examination of systems of power, social and built environments, individual responses to police violence, and physiological pathways linking police violence to health outcomes are needed. Notably, this review identified several public databases and validated questionnaires that can be used in future studies.

## Conclusions

The present review sought to map the definitions and measures of police violence in health literature. There is an increasing demand for research that investigates the impact of police violence on a range of health outcomes. However, a limited number of studies have explored how to conceptualize and operationalize this complex phenomenon. Our study makes a significant contribution to the existing literature on police violence and health by clarifying definitions and measures of police violence while also identifying critical gaps in current research. In this review, we identified three primary measures of police violence: self-reports, incident counts from existing databases, and medical records. To advance the understanding of police violence exposure, it is crucial for researchers to consistently refine their measurement approaches. This can be achieved by carefully operationalizing terms, utilizing psychometrically validated measures, and employing methodologies that shed light on the impact of police violence on health, especially among historically marginalized communities. Given that these communities face compounded effects from police violence and additional stressors related to systemic discrimination, it is vital to investigate how these factors influence health outcomes.

## Data Availability

All data generated or analyzed during this study are included in this published article and the publicly available protocol paper.

## Abbreviations

CDC: Centers for Disease Control and Prevention
CINAHL: Cumulative Index to Nursing and Allied Health Literature
EPPS: Expectation of Police Practice Scale
GVA: Gun Violence Archive
LGBTQ +: Lesbian, gay, bisexual, transgender and queer
ICD: International Classification of Diseases (ICD) codes
MeSH: Medical Subject Heading
M-CRFRE: Modified version of the Classes of Racism Frequency of Racial Experiences Measure
MPV: Mapping Police Violence
NVDRS: National Violent Death Report System
NVSS: National Vital Statistics System
PPI: Police Practices Inventory
WHO: World Health Organization
